# Disability and the risk of subsequent mortality in elderly: a 12-year longitudinal population-based study

**DOI:** 10.1186/s12877-021-02611-1

**Published:** 2021-11-23

**Authors:** Yang Yang, Zhaohui Du, Yafei Liu, Jiahui Lao, Xiaoru Sun, Fang Tang

**Affiliations:** 1 Department of Gerontology, The First Affiliated Hospital of Shandong First Medical University & Shandong Provincial Qianfoshan Hospital, Jinan, China; 2 Center for Big Data Research in Health and Medicine, The First Affiliated Hospital of Shandong First Medical University & Shandong Provincial Qianfoshan Hospital, Jinan, China; 3 Fred Hutchinson Cancer Research Center, Seattle, WA 98109 USA; 4 Department of Biostatistics, School of Public Health, Cheeloo College of Medicine, Shandong University, Jinan, China; 5 Shandong Provincial Qianfoshan Hospital, Cheeloo College of Medicine, Shandong University, No. 16766 Jingshi Road, Jinan, 250014 Shandong China

**Keywords:** Disability, Elderly, Cohort study, Death, Cox proportional hazards model

## Abstract

**Background:**

Assessment the impact of disability on mortality among the elderly is vital to healthy ageing. The present study aimed to assess the long-term influence of disability on death in the elderly based on a longitudinal study.

**Method:**

This study used the Chinese Longitudinal Healthy Longevity Study (CLHLS) data from 2002 to 2014, including 13,666 participants aged 65 years and older in analyses. The Katz ADL index was used to assess disability status and levels. Cumulative mortality rates were estimated by the Kaplan-Meier method. Cox proportional hazards models were conducted to estimate associations between disability and all-cause mortality for overall participants, two age groups as well as specific chronic disease groups. All reported results were adjusted by survey weights to account for the complex survey design.

**Results:**

During the 12-year follow-up, the death density was 6.01 per 100 person-years. The 3-years’ cumulative mortality rate of nondisabled elderly was 11.9% (95%CI: 10.9, 12.9%). As the level of disability increased, the cumulative mortality rate was from 28.1% (95%CI: 23.0, 33.1%) to 77.6% (95%CI: 63.8, 91.4%). Compared with non-disabled elderly, the multiple-adjusted hazard ratio of death due to disability was 1.68 (95% CI: 1.48, 1.90). The hazard ratios varied from 1.44 (95%CI: 1.23, 1.67) to 4.45 (95%CI: 2.69, 7.38) after classifying the disability levels. The hazard ratios of death in the young-old group (65–79 years) were higher than the old-old group (80 years and over) in both level B (HR = 1.58, 95%CI: 1.25, 2.00 vs. HR = 1.22, 95%CI: 1.06, 1.39, *P* = 0.029) and level G (HR = 24.09, 95%CI: 10.83, 53.60 vs. HR = 2.56, 95%CI: 1.75, 3.74, *P* < 0.001). For patients with hypertension, diabetes, heart disease, cerebrovascular disease as well as dementia, disability increases their relative risk of mortality by 1.64 (95%CI: 1.40, 1.93), 2.85 (95%CI: 1.46, 5.58), 1.45 (95%CI: 1.02, 2.05), 2.13 (95%CI: 1.54, 2.93) and 3.56 (95%CI: 1.22, 10.38) times, respectively.

**Conclusions:**

Disability increases the risk of all-cause death in the elderly, especially those with chronic diseases and the young-old group. Further studies are needed to better understand how to effectively prevent disability in the older population.

**Supplementary Information:**

The online version contains supplementary material available at 10.1186/s12877-021-02611-1.

## Introduction

Ageing is a worldwide issue and presents tremendous challenges. The World Population Ageing 2020 Highlights [1] showed that the total population was 727 million aged 65 years or over in 2020, and expected to reach over 1.5 billion in 2050. According to the latest 2021 census report by the National Bureau of Statistics of China [2], the number of Chinese elderly aged 60 and over has reached 264 million, accounting for 18.70% of the total population. Moreover, there were 190.64 million persons in the age group of 65 and over, accounting for 13.50%. With the increasing ageing of the population, the influence of disability among the elderly is becoming prominent. Previous studies have shown that disability prevalence increased with age [3–5], the rapid population ageing will inevitably increase the number of disabled people in China. Loss of independence in the activity of daily living (ADL) among the older adults not only leads to a decline in their physical and mental health but also imposes an enormous burden on their family as well as raises considerable challenges to the public health system [2].

Previous studies showed that disability in the elderly was higher and inevitable at the end of life [4–8]. The Fourth Survey on the Living Conditions of the Elderly in China reported that 18.3% of the older Chinese population were totally disabled or partially disabled in the year 2015 [6]. A retrospective study showed that the probability of disability in the last month of the decedent’s life exceeded 50% [4]. Given the severe ageing problem of China, understanding the impact of disability on mortality among the elderly is vital to healthy ageing [2].

However, there is a lack of evidence related to exploring the effects of disability status on death in the Chinese elderly. Besides, some studies used a dichotomous (yes or no) representing disabilities in activities of daily living [3, 7–9], ignoring the effects of different levels of disability. It can lead to an underestimation of the risk of death in the severely disabled population. Some studies showed that loss of independence complicated the care of people with chronic diseases and lower their quality of life [10, 11]. But little evidence to indicate disabling affects people with different chronic diseases. In the present study, we used the CLHLS data to explore the impact of disability on death among the Chinese elderly. In addition, we examined the effects of disability on older adults with specific chronic diseases.

## Methods

### Data source

Data was obtained from the CLHLS, a nationwide longitudinal study aimed at understanding factors related to healthy longevity in older Chinese population. Details of the study design, survey methodology, response rates and quality assessments of the data have been described in previous studies [12, 13].

The samples were randomly selected from 22 of 31 provinces in China, representing approximately 85% of the Chinese population. Eight waves of surveys were implemented in 1998, 2000, 2002, 2005, 2008, 2011, 2014 and 2018. In the 1998’s survey, CLHLS attempted to interview almost all centenarians. Researchers recruited approximately equal numbers of participants aged 80 years and over by random sampling design. Over-sampled were used to avoid the problem of small sub-sample sizes at the more advanced ages, especially for oldest-old males. In the 2002’s survey, the CLHLS added a sub-sample of older adults aged 65–79 in the same area [13]. All information was obtained through in-home face-to-face interviews, using internationally compatible questionnaires [14]. For older persons who were seriously ill or had cognitive impairs, their next of kin were interviewed.

**Fig. 1:**
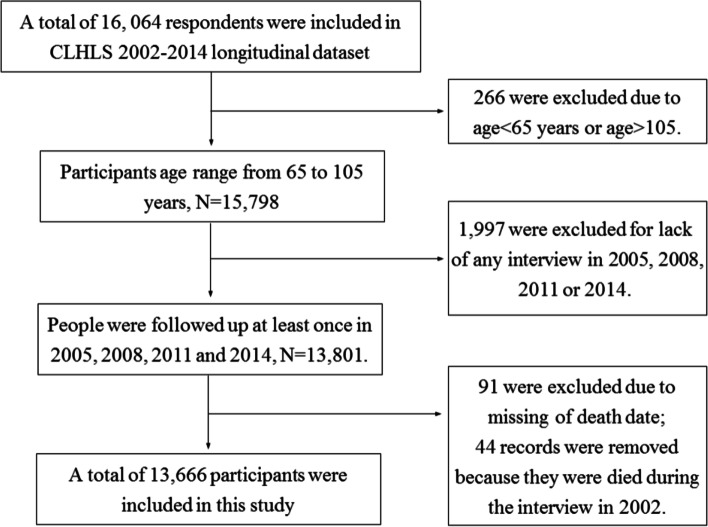
Flowchart of the study participants

The longitudinal data from 2002 to 2014 was used in this study. A total of 16,064 participants responded to the questionnaires. Individuals were excluded if they were younger than 65 or older than 106 years of age (*N* = 266) because of lack of sampling weights, had missing data in death date (*N* = 91), died at baseline year (*N* = 44) or lack of at least once interview in 2005, 2008, 2011 or 2014 (*N* = 1997). There were 13,666 participants (4133 young-old aged 65–79 years and 9736 old-old aged 80+) included for analysis (Fig. 1).

### Disability

Functional status of each participant was measured using the Katz index, which ranked adequacy of performance in the 6 functions of bathing, transferring, dressing, eating, toileting and continence [15]. Disability was defined as needing help with at least one of the six functions. The levels of disability were defined as follows [16]: Level-A indicated independence in all of the six functions; Level-B indicated independence in all but one of six functions; Level-C was independent in all but bathing and one additional function; Level-D was dependent on bathing, dressing and one other function; Level-E was dependent in bathing, dressing, toileting, and one additional functions; Level-F was dependent on bathing, dressing, toileting, transferring and one other function; Level-G was dependent on all six functions; Level-other was dependent in at least two functions and not classified as C, D, E or F.

### Death

Death information was collected from the official death certificates when available. Otherwise, the information was obtained from next-of-kin and local residential committees [14].

### Covariates

We included a variety of covariates based on literature review, including age, gender, socioeconomic, lifestyle and disease histories. Socioeconomic covariates combined marital status (categorized as married, widowed, divorced or never married), education (categorized as illiteracy, 1–6, 7–9, 10–12, 12+ years of schooling), place of residence (urban, rural) and co-resident status (live with family members, live alone, in an institution). Lifestyle covariates included smoking (never, current smoking, smoking cessation) and alcohol drinking (never, currently drinking, drinking cassation). Blood pressures were measured after sitting for 5 min. Hypertension was defined as systolic blood pressure ≥ 140 mmHg or diastolic blood pressure ≥ 90 mmHg or had a self-reported history of hypertension. Other chronic disease histories included diabetes, heart disease, Parkinson’s disease, dementia, cerebrovascular diseases and cancer were self-reported.

### Ethics approval

The CLHLS study was approved by the Research Ethics Committee of Peking University and Duke University (IRB00001052–13074). The informed consent was signed by the subject or a close relative [17]. All methods were performed in accordance with relevant guidelines and regulations.

### Statistical analysis

The characteristic of participants was strata by two age groups (65–79 yeas versus 80 years and over). Considering the over-sampled design approach in the CLHLS study, Sample weights were used to illustrate the complex survey design in all analyses [18, 19]. Proportions of variables were corrected by adjusting the sampling weights using the method by Zeng Y. et al. [14] when compared between age groups. The calculation formula was as follows.

$${w}^{\prime}\left(x,s,r\right)=w\left(x,s,r\right)\times \frac{T_j}{\sum_x{\sum}_r{\sum}_s\left[w\left(x,s,r\right)n\left(x,s,r\right)\right]}$$

*w*(*x*, *s*, *r*) was the age(x), sex(s), urban-rural residence(r) special weights provided in the CLHLS; *T*_*j*_ as the total number of interviewed persons of age group *j*; the *n*(*x*, *s*, *r*) was the number of persons of age *x*, sex *s*, and residence *r*.

Continuous variables were described as weight-adjusted means (± standard deviation [SD]) and compared using *t*-test. Categorical variables were described as percentages (%) and compared using the Rao-Scott chi-square test [19]. Cumulative mortality rates were estimated by the Kaplan-Meier method. Weighted Cox proportional hazards models [20] were used to examine the association between baseline disability and all-cause mortality. We also investigated associations between ADL disability levels and all-cause mortalities. The *z* statistic was used to test the difference in HRs between the age-group Cox models [21]. Besides, to assess the effect of disability on death among older adults with specific diseases, 7 sub-cohorts (hypertension, diabetes, cerebrovascular disease, heart disease, dementia, Parkinson or cancer at baseline) were constructed and Cox models were performed within each sub-cohort. All models were adjusted for baseline age, gender, education, family income level, marital status, smoking, drinking, residence, co-residence and chronic diseases. All analyses were conducted among overall samples as well as separately in the two age groups. All statistical analyses were performed using SAS software version 9.4 (SAS Institute, Inc., Cary, NC, USA).

## Results

During the study period, 9, 950 (72.81%) out of the 13, 666 older adults died. The median follow-up period was 4.8 ± 3.8 years (3.71 ± 2.95 years for individuals who died, 7.61 ± 4.18 years for those survived or lost follow-up). The overall death density of the elderly was 6.01 per 100 person-years (5.07 per 100 person-years in the young-old group and 10.79 per 100 person-years in the old-old group, *P* < 0.001) during 12 years of follow-up.

The characteristics of the subjects were described in Table 1. Compared to the young-old adults, the old-old group had statistically significantly larger proportions of females, illiteracy individuals and widows, higher prevalence of hypertension, diabetes and dementia (*P* < 0.001). The disability rate was 5.62% in the 65–79 age group and 21.21% in the old-old age group (*P* < 0.001). The distribution of disability levels (Katz index group) also statistically significantly differed between age groups (*P* < 0.001). The Kaplan-Meier survival curve (Supplement Fig. S1) by age group and disability status showed that disabled persons had lower survival rates within the same age group (*P* < 0.001, log-rank test). In particular, 20.73% of the young-old adults with disabled survived to the end of the 12-year follow-up period, while this proportion was only 5.51% in the old-old group with disability.

**Table 1:** Characteristics of Participants^a^

Variables	Overall	65–79 years	More than 80 years	***P***
	(***N*** = 13, 666)	(***N*** = 4, 133)	(***N*** = 9, 533)	
**Age, mean (SD)**	72.55 (0.07)	70.67(0.07)	83.73 (0.05)	< 0.001
**Sex (%)**				< 0.001
Male	47.58	50.36	28.48	
Female	52.42	49.64	71.51	
**Education level (%)**				< 0.001
Illiteracy	51.65	49.29	66.18	
1–6 years	36.23	37.83	26.50	
7–9 years	6.25	6.68	3.63	
10–12 years	3.85	4.10	2.32	
more than 12 years	2.01	2.11	1.38	
**Family income level(%)**				
< 1000 RMB	24.82	24.04	29.57	< 0.001
1000–4000 RMB	49.51	49.93	46.94	
> 4000 RMB	26.66	26.02	23.49	
**Marital status (%)**				< 0.001
Married	60.40	65.55	29.03	
Widowed	37.61	32.37	69.50	
Divorce or Never married	1.99	2.07	1.48	
**Residence (%)**				0.044
Urban	31.86	24.39	26.28	
Rural	68.14	75.61	73.72	
**Co-residence (%)**				< 0.001
With family member	85.70	86.91	78.30	
Live alone	12.36	11.72	16.23	
In an institution	1.94	1.37	5.43	
**Drink status (%)**				< 0.001
Never	66.61	66.40	67.94	
Drinking	24.05	24.74	19.85	
Drinking cessation	9.34	8.87	12.20	
**Smoke status (%)**				< 0.001
Never	60.35	59.24	67.14	
Smoking	26.10	27.59	17.08	
Smoking cessation	13.55	13.18	15.77	
**Chronic condition (%)**				
Hypertension	54.05	53.17	59.35	< 0.001
Diabetes	2.94	3.17	1.54	< 0.001
Heart disease	10.04	10.06	9.93	0.865
Cerebrovascular diseases	6.06	6.10	5.78	0.595
Parkinson’s disease	0.31	0.28	0.52	0.085
Dementia	0.56	0.37	1.77	< 0.001
Cancer	0.51	0.55	0.28	0.074
**Disability rate (%)**	7.82	5.62	21.23	< 0.001
**Katz index group (%)**				< 0.001
A	92.19	94.39	78.77	
B	5.09	4.08	11.23	
C	0.60	0.34	2.22	
D	0.37	0.24	1.16	
E	0.36	0.15	1.65	
F	0.53	0.35	16.4	
G	0.35	0.21	1.20	
Others	0.51	0.25	2.13	
**Death density(%)**	6.01	5.07	14.90	< 0.001

^a^Reported values were adjusted for survey weights to illustrate for the complex survey design

Table 2 showed cumulative mortality rates in the eight Katz index groups. The 3-years’ cumulative mortality rate for non-disabled elderly was 11.9% (95%CI: 10.9, 12.9%). As the level of disability increased, the cumulative mortality rate for overall older adults was from 28.1% (95%CI: 23.0, 33.1%) to 77.6% (95%CI: 63.8, 91.4%). Compared to overall older adults who were not disabled at baseline, those disabled persons had a 1.68 (95%CI: 1.48, 1.90)-fold increased hazard rate of all, cause death (Table 3). Those with Level-B disability had a 1.44 (95%CI: 1.23, 1.67)-fold increased hazard rate of all-cause death, and this association was stronger in people with Level-G disability (HR = 4.45, 95%CI: 2.69, 7.38). On average, each item disability increased in six-ADL functions was associated with a 1.26 (95% CI: 1.20, 1.32)-fold increase in all-cause mortality rate (data not shown). There was no statistical difference in the effect of disability (yes vs. no) on the risk of death among the elderly in both age groups (*P* = 0.189). However, the hazard risk of death was higher among the young-old elderly when one of six ADL functions (*P* = 0.029) or all six ADL functions loss (*P* < 0.001).

**Table 2:** Three-year’s cumulative mortality rates of baseline ADL index subgroups^a^

ADLs Index at baseline	3-year’s cumulative mortality rate (95%CI)		
	Overall	65–79 years	More than 80 years
A	11.9 (10.9, 12.9)	9.7 (8.7, 10.7)	28.1 (26.1, 30.1)
B	28.1 (23.0, 33.1)	22.7 (16.2, 29.1)	41.4 (36.1, 46.6)
C	47.2 (33.5, 60.9)	27.2 (5.5, 48.9)	65.6 (55.2, 76.1)
D	49.1 (31.3, 66.9)	45.9 (16.8, 74.9)	53.2 (36.3, 70.1)
E	55.1 (38.0, 72.1)	46.1 (8.6, 83.6)	64.3 (52.4, 76.2)
F	54.4 (38.9, 69.9)	43.8 (19.0, 68.6)	73.3 (61.5, 85.2)
G	77.6 (63.8, 91.4)	87.3 (65.8, 100.0)	75.0 (63.6, 86.5)
Others	47.9 (30.7, 65.0)	28.7 (0, 61.4)	65.6 (53.1, 78.1)

Abbreviations: *CI* confidence interval

^a^Reported values were adjusted for survey weights to illustrate for the complex survey design

**Table 3:** Association of disability status with mortality in the whole population and different age groups

Disability status at baseline	Overall		65–79 years		More than 80 years		***P*** for difference between age groups
	Death/Survival (9917/3712)	HR (95%CI) ^**a**^	Death/Survival (1759/2372)	HR (95%CI) ^**a**^	Death/Survival (8158/1340)	HR (95%CI) ^**a**^	
**Disability**							
No	6324/3297	Reference	1589/2274	Reference	4735/1023	Reference	
Yes	3593/415	1.68 (1.48, 1.90)^***^	170/98	1.74 (1.42, 2.14) ^***^	3423/317	1.57 (1.40, 1.76) ^***^	0.189
**ADLs Index**							
A	6324/3297	Reference	1589/2274	Reference	4735/1023	Reference	
B	1486/277	1.44 (1.23, 1.67) ^***^	109/81	1.58 (1.25, 2.00) ^***^	1377/196	1.22 (1.06, 1.39) ^**^	0.029
C	475/41	2.16 (1.52, 3.07) ^***^	12/7	1.77 (0.87, 3.59)	463/34	2.55 (2.08, 3.11) ^***^	0.166
D	204/14	2.45 (1.53, 3.90) ^***^	11/2	2.57 (1.19, 5.54) ^*^	193/12	2.11 (1.45, 3.08) ^***^	0.329
E	307/23	2.33 (1.55, 3.51) ^***^	6/1	2.21 (0.99, 4.91)	301/22	2.34 (1.53, 3.57) ^***^	0.451
F	386/16	2.71 (1.95, 3.75) ^***^	16/2	3.15 (1.76, 5.61) ^***^	370/14	2.29 (1.61, 3.26) ^***^	0.179
G	350/12	4.45 (2.69, 7.38) ^***^	10/0	24.09 (10.83, 53.60) ^***^	340/12	2.56 (1.75, 3.74) ^***^	< 0.001
Others	385/32	1.73 (1.14, 2.61) ^**^	6/5	1.09 (0.43, 2.79)	379/27	2.17 (1.50, 3.12) ^***^	0.091

Abbreviations: *ADLs* activities of daily living, *HR* hazard ratio, *CI* confidence interval

^a^Multivariate model tests are adjusted for age, sex, education, family income level, marital status, smoke, drink, residence, co-residence, hypertension, diabetes, cerebrovascular diseases, dementia, Parkinson’s and cancer using the survey weights

^***^P < 0.001

^**^P < 0.01

^*^P < 0.05

**Fig. 2:**
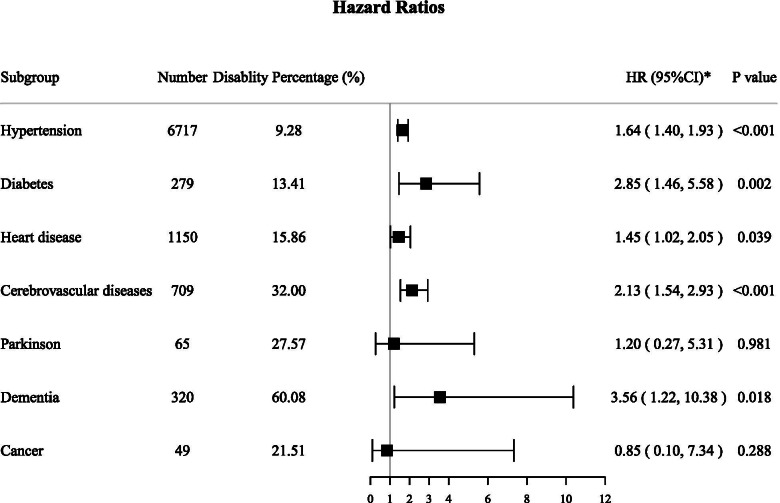
The influence of disability status on the future death of patients with chronic disease ^a^. Abbreviations: HR, hazard ratio; CI, confidence interval. ^a^ In all subgroups, non-disabled patients were used as reference. Adjusted for age, sex, education, family income level, marital status, smoke, drink, residence, co-residence, and other chronic diseases besides this subgroup of diseases using the survey weights

Figure 2 shows the effect of disability on death among the 7 chronic disease sub-cohorts. After adjusted for survey weights and covariate variables, disability increased the risk of death in elderly patients with hypertension, diabetes, heart disease, cerebrovascular disease, and dementia (*P* < 0.05). Specifically, disability raised hypertension patients’ death risk by 1.64 (95%CI: 1.40, 1.93) times, diabetes patients’ by 2.85 (95%CI: 1.46, 5.58) times, heart disease patients’ by 1.45 (95%CI: 1.02, 2.05) times, cerebrovascular disease patients’ by 2.13 (95%CI: 1.54, 2.93) and 3.56 (95%CI: 1.22, 10.38) times in dementia patients.

## Discussion

The key findings from this prospective study of old Chinese were as follow: (1) accompanying disability in ADLs had a strong impact on mortality rates raise in both the young-old and old-old groups. (2) Compared with the old-old group, a higher death risk was observed among the young-old group when the elderly had one of six or all six ADLs functions disabled. (3) For the elderly who suffered from chronic diseases, disability further increased the risk of death.

Consistent with previous studies [10], we found that disability was an important risk factor for the death of the elder. A population-based study of British people over 50 reported disability relative risk of death was from 1.36 to 2.20 [7], slightly higher than our results. This distinction is due to the different definitions of disability. The British study used a comprehensive definition of disability, including physical injury (e.g. eyesight, hearing and arthritis), activities limitation (ADLs), and instrumental activities of daily living. By contrast, our study focused on the effects of daily living function on elder death. It was conducive to promote research on the prevention of motor dysfunction in the elder. Although no more than 1 in 10 people were disabled at baseline, the excess risk of death was 29.89% higher than those without. The value of this attributable risk was as high as 43.62% in the elderly over 80 years old. Consistent with the result of Pongiglione B et al. [7], as the Katz index increased, there was a clear hazarded tendency of death. Lack of daily living ability has both physiological and psychological effects on the elderly. Several studies [22] showed that disability was not only declining in people’s ability to live but also amplified an individual’s fear of death. Extra care and financial burden on families due to disability can also deepen depression and other negative emotions among the elderly [22, 23], which are important risk factors for occurring adverse events. Therefore, many people are afraid of living with disability, especially the disabled for a long time pre-death, rather than death itself.

Our study showed that even if only one of the daily activities was impaired, the 3-years cumulative mortality in the elderly was as high as 28.1% (95%CI: 23.0, 33.1%). Even in the young-old group, the 3-years cumulative mortality rate reached 22.7% (95%CI: 16.2, 29.1%). Unfortunately, Thomas M. Gill’s study [5] showed that it seemed inevitable to disable before death and no predictable trajectory for the disability process in the last year of life. This conclusion posed a huge challenge for the rational division of resources to care for the elderly at the end of their lives, especially in ageing countries. According to the results of the Cox regression model, the G level had 4.45 times death risk than those non-disabled elderly. In this study, the percentage of death attributed to disability state in the elderly was 5.04%, 3.99% in the young-old group and 10.79% in the old-old group (*P* < 0.001). It indicated that there was about 4 of every 100 elderlies can avoid death each year if effective prevention measures on disability were implemented.

The explanation for the correlation that disability in the elderly increased the risk of all-cause dying is multifactorial. On the one hand, age-related frailty was an important factor in physical disability and mortality [8, 24, 25]. Complex interactions such as muscle strength decline [24], inflammation [26], neurological disorder [27] and chronic diseases [28] in the elderly lead to disability, and these risk factors were also accelerating the occurrence of death [24]. On the other hand, multi-morbidity was inextricably linked to death and disability in older adults [29]. Further, physical frailty and loss of independence can complicate the care for chronically ill patients and lead to poor prognosis [30]. Our findings suggested that for older patients with chronic diseases, the inability to live independently increased the risk of death, which was consistent with the reports from North America and Europe [10, 31]. A study of heart failure patients in Canada [10] showed that disability increases the risk of death for heart failure patients by 1.49 to 2.26 times. According to a French study, dementia patients were disabled for approximately half of the period during the disease. Our study found that disability even increased 3.56 times mortality hazard among dementia patients. Although the complex network of chronic diseases and disabilities was unclear, the finding of a heavy health burden due to disability in elders called to implement prevention of disability among elders with chronic diseases. The relationships between disability and death in Parkinson’s and cancer patients were not observed, which may be caused by insufficient of sample size.

A population-based cohort study with more than 10-years’ follow-up [9] showed that once the ability of daily living of young patients was impaired, their expected duration of survival changed from twice to once than older patients. This was similar to our study’s finding. Although the cumulative mortality rate of the old-old disabled group was higher than the younger one, after adjusted age, fall history, cerebrovascular and other major confounding factors, the Cox model showed that it was opposite on the two groups’ hazard risk of death due to disability when only one of six or all six ADL functions disabled. However, this difference was non-significant when disability was classified as dichotomous. An ageing research review [32] pointed that as age growing, people’s diminished ability to respond to resistant illness, leading to poor health and ultimately death. Only those with higher intrinsic capacity can achieve longevity [33]. Unfortunately, few people in this group.

This study had both strengths and limitations. The strengths included: (1) we detailed the classification of ADL disability by number and components to assess the impact on mortality after adjusting sampling weights, which had rarely been taken into account in previous reports despite its importance for subjects with disability and significantly overexposed to death risk. (2) Moreover, we additionally analyzed the impact of disability on mortality in older adults with common chronic conditions, which had important implications for targeted preventive measures in primary care. (3) Finally, the sample of incident cases of disability was population-based and covered a 12-year follow-up period. There were limitations to be acknowledged. Underreporting was unavoidable in the self-report questionnaire studies, which led to misclassification of chronic disease and disabling levels in some populations. Although the Cox model adjusted for a number of variables, there were inevitably unobservable confounding factors. Therefore, the findings needed to be treated with caution. Although the CLHLS study design used weighted sampling, the original study design focused on the oldest-old and the sampling of the young-old group was insufficient. However, it was no denying that the demographic characteristics of our cohort did reflect the majority of older Chinese [3].

## Conclusions

In conclusion, this large population-based study highlighted the fact that disability, regardless of level, increased the risk of death in older adults, especially those who already suffered from chronic diseases. Moreover, compared with the old-old group, the young-old group had a lower mortality rate but a higher risk of death when ADL function disabled occurred. Health management strategies need to focus on initiatives to personalize health care and adapt to the long-term service demand of elderly with different disability statuses. In addition, interventions that prevent disability are needed to reduce the risk of death among the elders.

## Supplementary Information

**Additional file 1: Supplement Figure S1.** The Kaplan-Meier survival curve by age group and disability status.

## Data Availability

The datasets analyzed during the current study are available in the Peking University Open Research Data repository, https://opendata.pku.edu.cn/. The datasets are also available at https://www.icpsr.umich.edu/web/NACDA/series/487.

## Abbreviations

ADL: Activities of daily living
CLHLS: The Chinese Longitudinal Healthy Longevity Study
N: Number
HR: Hazard ratio
CI: Confidence interval

## References

[1] United Nations Department Of Economic And Social Affairs, Population Division. World Population Ageing 2020 Highlights: Living arrangements of older persons 2020. https://www.un.org/development/desa/pd/. Accessed 8 Jun 2021.

[2] National Bureau of Statistics of China. National Bureau of Statistics of China Statistical Communique of the People’s Republic of China on the 2020 National Economic and social development. http://www.stats.gov.cn/tjsj/zxfb/202102/t20210227_1814154.html. Accessed 24 May 2021.

[3] Liu Z, Han L, Wang X, Feng Q, Gill TM (2018). Disability prior to death among the oldest-old in China. J Gerontol A Biol Sci Med Sci.

[4] Smith AK, Walter LC, Miao Y, Boscardin WJ, Covinsky KE (2013). Disability during the last two years of life. JAMA Intern Med.

[5] Gill TM, Gahbauer EA, Han L, Allore HG (2010). Trajectories of disability in the last year of life. N Engl J Med.

[6] Dang W, Wei Y, Liu N (2018). Survey report on the living conditions of China’s urban and rural older persons (2018).

[7] Pongiglione B, De Stavola BL, Kuper H, Ploubidis GB (2016). Disability and all-cause mortality in the older population: evidence from the English longitudinal study of ageing. Eur J Epidemiol.

[8] Wu LW, Chen WL, Peng TC, Chiang ST, Yang HF, Sun YS (2016). All-cause mortality risk in elderly individuals with disabilities: a retrospective observational study. BMJ Open.

[9] Delva F, Touraine C, Joly P, Edjolo A, Amieva H, Berr C (2016). ADL disability and death in dementia in a French population-based cohort: new insights with an illness-death model. Alzheimers Dement.

[10] Dunlay SM, Manemann SM, Chamberlain AM, Cheville AL, Jiang R, Weston SA (2015). Activities of daily living and outcomes in heart failure. Circ Heart Fail.

[11] Fong JH (2019). Disability incidence and functional decline among older adults with major chronic diseases. BMC Geriatr.

[12] Liu Z, Han L, Feng Q, Dupre ME, Gu D, Allore HG (2019). Are China’s oldest-old living longer with less disability? A longitudinal modeling analysis of birth cohorts born 10 years apart. BMC Med.

[13] Zhang Q, Wu Y, Han T, Liu E (2019). Changes in cognitive function and risk factors for cognitive impairment of the elderly in China: 2005-2014. Int J Environ Res Public Health.

[14] Zeng Y, Feng Q, Hesketh T, Christensen K, Vaupel JW (2017). Survival, disabilities in activities of daily living, and physical and cognitive functioning among the oldest-old in China: a cohort study. Lancet..

[15] Arik G, Varan HD, Yavuz BB, Karabulut E, Kara O, Kilic MK (2015). Validation of Katz index of independence in activities of daily living in Turkish older adults. Arch Gerontol Geriatr.

[16] KATZ S, FORD AB, MOSKOWITZ RW, JACKSON BA, JAFFE MW (1963). Studies of illness in the aged. The index of Adl: a standardized measure of biological and psychosocial function. JAMA..

[17] Lv X, Li W, Ma Y, Chen H, Zeng Y, Yu X, Hofman A, Wang H. Cognitive decline and mortality among community-dwelling Chinese older people. BMC Med. 2019;17(1):63. 10.1186/s12916-019-1295-8 PMC6419492 30871536

[18] Zeng Y, Vaupel JW, Xiao Z, Zhang C, Liu Y (2001). The healthy longevity survey and the active life expectancy of the oldest old in China. Popul Eng Select.

[19] Lohr SL (2000). Sampling: design and analysis. Technometrics..

[20] David A (1992). BINDER, Fitting Cox's proportional hazards models from survey data. Biometrika..

[21] Liu J, Hong Y, D’Agostino RB, Wu Z, Wang W, Sun J (2004). Predictive value for the Chinese population of the Framingham CHD risk assessment tool compared with the Chinese multi-provincial cohort study. JAMA..

[22] Read JR, Sharpe L, Modini M, Dear BF (2017). Multimorbidity and depression: a systematic review and meta-analysis. J Affect Disord.

[23] Lo Monaco M, Mallaci Bocchio R, Natoli G, Scibetta S, Bongiorno T, Argano C (2020). Human relationships in patients’ end-of-life: a qualitative study in a hospice ward. Intern Emerg Med.

[24] Lavie CJ, Ozemek C, Carbone S, Katzmarzyk PT, Blair SN (2019). Sedentary behavior, exercise, and cardiovascular health. Circ Res.

[25] Afilalo J, Alexander KP, Mack MJ, Maurer MS, Green P, Allen LA (2014). Frailty assessment in the cardiovascular Care of Older Adults. J Am Coll Cardiol.

[26] Friedman E, Shorey C (2019). Inflammation in multimorbidity and disability: an integrative review. Health Psychol.

[27] Mat Rosly M, Mat Rosly H, Davis Oam GM, Husain R, Hasnan N (2017). Exergaming for individuals with neurological disability: a systematic review. Disabil Rehabil.

[28] GBD 2017 Risk Factor Collaborators (2018). Global, regional, and national comparative risk assessment of 84 behavioural, environmental and occupational, and metabolic risks or clusters of risks for 195 countries and territories, 1990–2017: a systematic analysis for the Global Burden of Disease Study 2017. Lancet (London, England).

[29] Dugravot A, Fayosse A, Dumurgier J, Bouillon K, Rayana TB, Schnitzler A (2020). Social inequalities in multimorbidity, frailty, disability, and transitions to mortality: a 24-year follow-up of the Whitehall II cohort study. Lancet Public Health.

[30] Schoeller SD, Lima DKS, Martins MM, Ramos FRS, Zuchetto MA, Bampi LNDS (2018). Protocol for a scoping review on nursing care and the autonomy of disabled persons. BMJ Open.

[31] Bleijenberg N, Zuithoff NPA, Smith AK, De Wit NJ, Schuurmans MJ (2017). Disability in the individual ADL, IADL, and mobility among older adults: a prospective cohort study. J Nutr Health Aging.

[32] Gonzalez-Freire M, Diaz-Ruiz A, Hauser D, Martinez-Romero J, Ferrucci L, Bernier M (2020). The road ahead for health and lifespan interventions. Ageing Res Rev.

[33] Beard JR, Officer A, de Carvalho IA, Sadana R, Pot AM, Michel JP (2016). The world report on ageing and health: a policy framework for healthy ageing - ScienceDirect. Lancet..

