# Supplementary material for: Disability and the risk of subsequent mortality in elderly: a 12-year longitudinal population-based study
Source: BMC Geriatr. 2021 Nov 23;21:662. doi: 10.1186/s12877-021-02611-1 (PMC8609873; doi:10.1186/s12877-021-02611-1)
Supplement: Supplementary file 1 — Additional file 1: Supplement Figure S1. The Kaplan-Meier survival curve by age group and disability status. [file 12877_2021_2611_MOESM1_ESM.pdf]

Title: Disability and the risk of subsequent mortality in elderly: A 12-year longitudinal population-based study

Supplement Figure S1: The Kaplan-Meier survival curve by age group and disability status

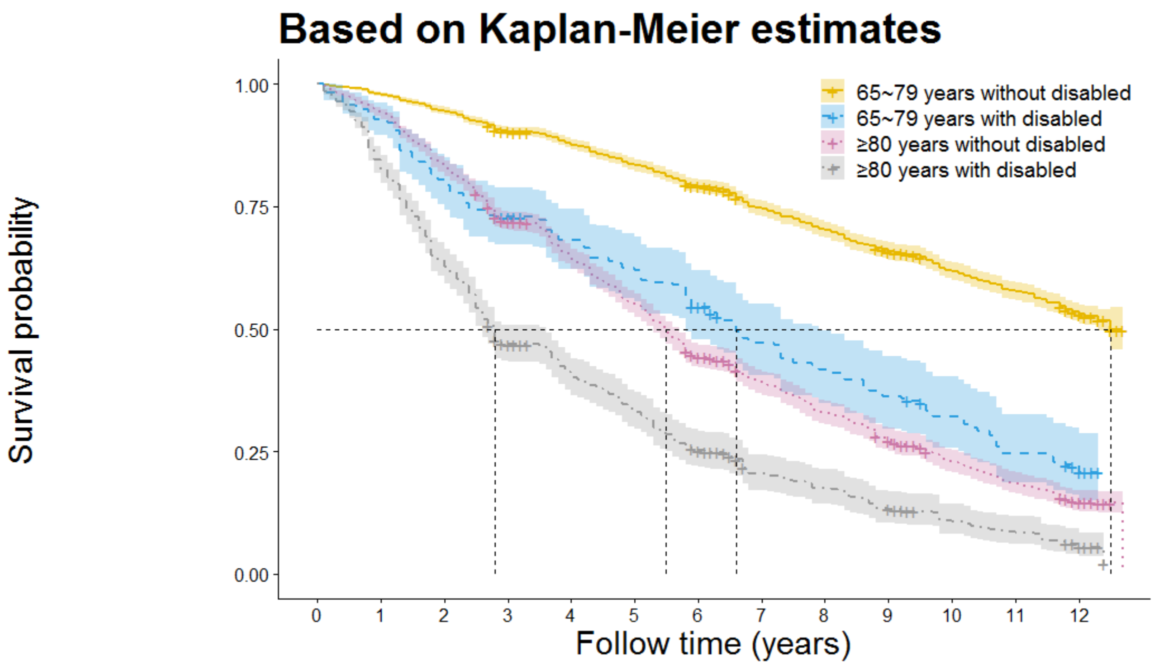

Number at risk

Follow time (years)	0	1	2	3	4	5	6	7	8	9	10	11	12
65~79 years without disabled	11043	10826	10458	9222	8184	7786	7306	6267	5908	5542	4955	4620	3271
65~79 years with disabled	658	610	529	417	344	315	270	210	185	161	132	101	73
≥80 years without disabled	1511	1426	1265	1015	829	705	564	439	370	309	243	197	122
≥80 years with disabled	407	344	261	177	140	115	86	62	53	41	31	25	16
